# Surgical outcomes of sinonasal inverted papilloma: a 17 year review^☆^

**DOI:** 10.1016/j.bjorl.2018.12.011

**Published:** 2019-02-21

**Authors:** Gil Coutinho, Joana Marques, Manuel Leal, Jorge Spratley, Margarida Sá Fernandes, Margarida Santos

**Affiliations:** a Centro Hospitalar São João, Departamento de Otorrinolaringologia, Porto, Portugal; b Faculdade de Medicina da Universidade do Porto, Unidade de Otorrinolaringologia, Porto, Portugal; c Centro de Investigação em Tecnologia e Serviços de Saúde (CINTESIS), Porto, Portugal; d Centro Hospitalar São João, Departamento de Patologia, Porto, Portugal; e Universidade do Porto, Faculdade de Medicina, Departamento de Patologia, Porto, Portugal

**Keywords:** Inverted papilloma, Endoscopy, Treatment outcome, Recurrence, Papiloma invertido, Endoscopia, Resultado do tratamento, Recorrência

## Abstract

**Introduction:**

Inverted papillomas represent one of the most common benign neoplasic lesions located in the sinonasal tract. Owing to the local erosive behavior, tendency to recur and the potential for malignant transformation, surgical management of inverted papillomas is often challenging.

**Objective:**

This study aimed to analyze the surgical outcomes of patients with inverted papillomas, according to the Krouse staging and the different surgical approaches.

**Methods:**

Retrospective study of patients diagnosed with sinonasal inverted papillomas who underwent surgical treatment between 2000 and 2016 at a tertiary referral hospital. Cases with follow-up less than 12 months were excluded. The rate and the time of recurrence were the main outcomes. Values of *p* < 0.05 were considered statistically significant.

**Results:**

Thirty-six cases with mean age of 60 years, predominantly male (72%), were included. The follow-up period ranged from 1 to 16 years, with an average of 4.5 years. Krouse T1 Stage corresponded to 11.1%; T2 occurred in 50% of cases; while T3 and T4 Stages accounted for 30.6% and 8.3% of patients, respectively. Most cases were approached by an endoscopic technique alone (83.3%), with a recurrence rate of 13.3%. Patients treated via a combined or open approach revealed a recurrence of 16.7%. No differences in the recurrence rate were reported when comparing endoscopic surgery with the open or combined techniques. Krouse Stage T3 had a significant association with inverted papillomas recurrence (*p* = 0.023). All inverted papilloma relapses occurred up to 2 years post-operatively. One case of malignant transformation was recorded (2.7%).

**Conclusion:**

Endoscopic surgery did not increase the recurrence rates and can be a safe and efficient alternative to open or combined techniques. The recurrence of inverted papillomas seem to be related to the persistence of the disease and tend to occur early after primary surgery. Krouse T3 Stages may be associated with a higher recurrence of inverted papillomas.

## Resumo

**Introdução:**

Os papilomas invertidos são uma das lesões neoplásicas benignas mais comuns no trato nasossinusal. Devido ao seu comportamento localmente agressivo, tendência para recidivar e potencial de malignização, o tratamento cirúrgico dos papilomas invertidos constitui frequentemente um desafio.

**Objetivo:**

Analisar os resultados cirúrgicos dos papilomas invertidos segundo o estadiamento de Krouse, bem como avaliar as diferentes abordagens cirúrgicas.

**Método:**

Estudo retrospectivo de pacientes com diagnóstico de papiloma invertido nasossinusal submetidos a tratamento cirúrgico entre 2000 e 2016 em hospital terciário. Casos com acompanhamento inferior a 12 meses foram excluídos. A taxa e o tempo de recidiva foram os principais resultados analisados desfechos avaliados. Valores p 0,05 foram considerados estatisticamente significativos.

**Resultados:**

Foram incluídos 36 casos, com média de idade de 60 anos, predominantemente do sexo masculino (72%). O período de acompanhamento variou de 1 a 16 anos, em uma média de 4,5 anos. Relativamente ao estadiamento, 11,1% dos pacientes foram classificados como estadio Krouse T1, 50% como T2, 30,6% como T3 e 8,3% como T4. A maioria dos casos foi tratada exclusivamente por cirurgia endoscópica (83,3%), com taxa de recidiva de 13,3%. Pacientes tratados com uma técnica combinada ou aberta apresentaram recidiva de 16,7%. Não foram observadas diferenças quanto à taxa de recidiva entre os casos abordados por via endoscópica e os casos tratados com técnica aberta ou combinada. Verificou-se uma associação significativa entre o estadio T3 de Krouse e recidiva de papilomas invertidos (*p* = 0,023). Todas as recidivas de papilomas invertidos foram observadas até dois anos após a cirurgia. Um caso de transformação maligna foi registrado (2,7%).

**Conclusão:**

A cirurgia endoscópica não aumentou as taxas de recidiva e pode ser uma alternativa segura e eficiente às técnicas abertas ou combinadas. Os casos de recidiva do papiloma invertido parecem estar relacionados com a persistência da doença e tendem a ocorrer precocemente após a cirurgia primária. Os estadios T3 de Krouse podem estar associados a uma maior recidiva.

## Introduction

Inverted Papillomas (IP) originate from the schneiderian epithelium^1^ and represent one of the most common benign neoplasic lesions located in sinonasal tract,^2^, ^3^ with an incidence between 0.2 and 1.5 cases per 100,000 persons annually,^4^, ^5^, ^6^ and with a male-to-female ratio of 3.4:1.^7^

The morphological features and clinical characteristics of IP have been well described, whereas its etiology and risk factors remain controversial. Certain hypotheses have been suggested, but causality has never been fully established for the suspected factors: smoking, allergy or occupational exposures.^5^ Recurrence and carcinomatous potential have for many years suggested a possible viral origin. However, a recent study from Mohajeri et al.^8^ does not support the role of the human papillomavirus (HPV) as an etiological factor for IP occurrence, neither for its progression to squamous cell carcinoma (SCC). In fact, among the 76 IP specimens, only 13% were HPV positive, whereas HPV was not detected in any of the SCC cases.^8^

The preoperative radiological assessment is of utmost importance to determine the tumor extension and its location. Computed tomography (CT) scans typically reveal a lesion within the middle meatus, associated with heterogeneous opacification and sclerosis in the adjacent bone.^9^ Focal areas of hyperostosis demonstrated on CT scans may correspond to the tumor implantation site in nearly 90% of cases.^10^ On magnetic resonance (MR) T1-weighted images, the tumor shows an aspect of cerebriform circumvolutions, typical of IP; the same aspect can be found on T2-weighted sequences, strongly favoring an IP diagnosis.^11^

Despite the recognized value of staging systems in assessing treatment methods for IP, there is currently no universally accepted system. Amongst the proposed staging systems the Krouse classification^12^ – which focuses on the extent of IP – has been widely used due to its ease of implementation and reproducibility.

Owing to the local erosive behavior, tendency to recur and the potential for malignant transformation,^13^ many surgeons recommended an open surgical approach for the treatment of advanced cases of IP.^4^, ^14^ However, the endoscopic technique for IP treatment seems to provide excision control rates comparable to the traditional open methods such as lateral rhinotomy and external frontoethmoidectomy.^3^

This study aimed to analyze the surgical results of IP, according to the Krouse staging and the different surgical approaches. The rate and the time of recurrence were the main outcomes.

## Methods

A retrospective study of patients diagnosed with sinonasal IP who underwent surgical treatment between January 2000 and December 2016 at a tertiary referral hospital was conducted. Cases with follow-up less than 12 months were excluded. Demographic, clinical, surgical and radiological data were retrospectively collected. All cases underwent preoperative CT scan which was complemented by MR for the more extended lesions. Surgical approaches were divided in two groups: (A) exclusively endoscopic and (B) open or combined techniques. The final diagnosis was established by histopathological examination. All patients were retrospectively staged using clinical, radiological and histopathological evaluations according to Krouse staging system (Table 1).

**Table 1:** Krouse staging system for inverted papilloma.

T1	Confined to the nasal cavity
T2	Involves ostiomeatal complex region, ethmoid, or medial wall of the maxillary sinus
T3	Involves any wall of the maxillary sinus but medial, frontal sinus, or sphenoid sinus
T4	Any extranasal or extrasinus extension or presence of a malignant neoplasm

Statistical analysis was performed using SPSS version 23.0 for macOS (SPSS Inc., Chicago, USA). The Chi-Squared test or the Fisher's exact test were applied when appropriate, to evaluate the statistical significance of the association between the categorical variables and recurrence. Multiple logistic regression analysis was used to evaluate the association between significant variables of the univariate analysis and recurrence. The time for recurrence was analyzed by a Kaplan–Meier survival curve. Values of *p* < 0.05 were considered statistically significant. The confidence intervals were set at a 95% level.

The current study met the approval of the Institutional Review Board (approval no. 09/18).

## Results

Thirty-six cases with a mean age of 60 years and a male predominance (72.2%) were included. The follow-up period ranged from 1 to 16 years, with an average of 4.5 years.

The most common site of origin of IP was the maxillary sinus (*n* = 16). Among these patients, nine involved the anterior, inferior or lateral maxillary sinus wall. The ethmoidal sinus and the nasal cavity were also common involved sites (Table 2).

**Table 2:** Number of cases and recurrences according to the implantation zone. A Chi-Squared test was conducted to assess any differences.

Site	No.	Recurrence (%)	*p*
Nasal cavity	10	0 (0)	–
Maxillary sinus	16	4 (25)	0.149
Ethmoid sinus	12	0 (0)	–
Frontal sinus	5	1 (20)	0.549
Sphenoid sinus	2	0 (0)	–

*Note*: Some tumors involved more than one anatomical site.

A pre- or intra-operative histological diagnosis of IP was established in 16 (44.4%) cases. Other benign histologies such as inflammatory polyps were diagnosed in 23.5% of cases who underwent a preoperative biopsy. The rate of recurrence did not differ between patients with a pre- or intra-operative IP diagnosis when compared to IP diagnosed during the postoperative period by histopathology (*p* = 0.654).

Krouse T1 Stage corresponded to 11.1%; T2 occurred in 50% of cases; while T3 and T4 stages accounted for 30.6 and 8.3% of patients, respectively (Table 3).

**Table 3:** Number of cases and recurrences according to Krouse staging system. A multiple logistic regression analysis was performed if recurrence occurred.

Krouse stage	No. (%)	Recurrence (%)	*p* (Odds Ratio)
T1	4 (11.1)	0 (0%)	–
T2	17 (50.0)	1 (5.9%)	0.179 (0.21)
T3	7 (30.6)	4 (57.1%)	0.029 (13.7)
T4	3 (8.3)	0 (0%)	–

Most of the cases were treated by endoscopic technique alone (83.3%), which composed our Group A. Under endoscopic control, the bulky exophytic tumor was resected by forceps or a microdebrider until the origin or base of the tumor was identified. The tumor base was managed either by en bloc resection or by piecemeal resection with careful handling of the tumor implantation site with drilling and bipolar cautery. The resected and the debrided tissue preserved in suction traps were sent for histological examination.

The Group B was composed by three types of patients according to the external approach used: (a) three patients were treated by a Caldwell-Luc technique for resection of tumors of the anterolateral region of the maxillary sinus; (b) a case of a frontoethmoid IP with lateral extension was removed via a lateral rhinotomy approach; (c) a bicoronal approach with frontal osteoplastic flap was the selected technique in two cases of anterior skull base erosion with intracranial invasion, in cooperation with neurosurgery. In one of these cases, owing to the extensive frontoethmoid involvement, the surgical approach was further extended with a lateral rhinotomy. After IP ressection, the exposed bone in the implantation area was routinely removed by drilling.

Regarding patients with advanced Krouse staging (T3 and T4), these comprised 27% of the endoscopically treated (Group A) and 100% of the patients who underwent an open or combined surgery (Group B) (Table 4).

**Table 4:** Number of cases and recurrences according to the surgical groups: Group A corresponding to endoscopically treated patients; while cases treated via combined or open approach belonged to Group B. Advanced cases comprised those with Krouse T3 or T4 IP. A Chi-Squared test was conducted to assess any differences.

Group	No.	Recurrence (%)	*p*
A	30	4 (13.3)	1.000
B	6	1 (16.7)	
Advanced A^a^	8	3 (37.5)	0.580
Advanced B^a^	6	1 (16.7)	
Total	36	5 (13.9)	–

a Only T3 or T4 Krouse disease.

Epistaxis requiring sphenopalatine artery ligation occurred in one case from Group A; whereas in Group B one patient developed a frontal mucocele and another one a postoperative epiphora.

**Figure 1:**
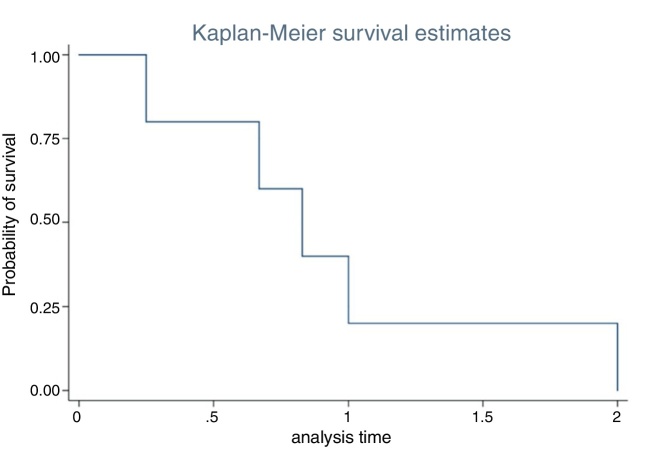
Kaplan–Meier survival analysis of time of recurrence in years. Note that all recurrences occurred up to two years after surgery.

The overall recurrence rate (Groups A and B) was 13.9%. One single case (2.7%) of synchronous malignant transformation was recorded and was treated with radiation therapy (RT) after surgery. All recurrences occurred within the first two years postoperatively, with a mean time of 11.4 months (Fig. 1). Only T3 Stage patients showed a significant association with IP recurrence, with a rate of 57.1% (Odds Ratio = 13.7; *p* = 0.029) (Table 3).

Concerning the surgical approach, cases from Group A presented a recurrence rate of 13.3% which compared to Group B recurrence of 16.7%. This difference was not statistically relevant (*p* = 1.000). Considering only cases of advanced Krouse staging (T3 and T4), the recurrence rates between Groups A and B continued not to differ (*p* = 0.580) (Table 4).

## Discussion

Thirty-six cases of IP were diagnosed and surgically treated at our institution during a 17 year period. Our institution's area of direct influence encompasses about 330,000 inhabitants, which corresponds to an estimated annual incidence of 0.64 cases per 100,000 with a male predominance of 2.6/1 and a mean age of 60 years. Epidemiologically, the current series of IP presented comparable results to preceding studies.^4^, ^5^, ^6^, ^7^

Also in accordance with other reports,^14^, ^15^, ^16^ IP arising from the maxillary sinus, nasal cavity and ethmoidal sinus were the most frequently found, whereas its exclusive location within the frontal or sphenoid sinus was exceedingly rare.

Pathologic examination is essential to reach a definite diagnosis and to exclude concomitant malignancy.^17^ However, pre- and intra-operative histological evidence of IP is not always feasible as IP may coexist with an inflammatory process. This accounts for false negative rates of up to 17% on biopsy as reported by Han and colleagues.^18^ In our study 23.5% of cases who underwent a preoperative biopsy were misdiagnosed with another benign pathology, most of them with inflammatory polyps. Despite the high false negative rate and the low proportion of pre- or intraoperative IP diagnosis (44.4%) recurrence rates were not different from those cases diagnosed during the postoperative time. This could be related to the high index of suspicion when addressing an unilateral insidious sinonasal neoplastic lesion with concurrent radiological signs such as areas of focal hyperostosis on CT scan, and the typical cerebriform circumvolutions which may be evident on MR. In this context, radiologic imaging with CT and mainly MR plays an important role in guiding the diagnosis and the extension of the IP.

The present study supports endoscopic surgery as a very effective treatment for patients with IP. The calculated recurrence rate was 13.3% without significant differences between the endoscopically treated and patients treated via a combined or open surgery. In the published literature recurrence rates vary widely from 0% to 50%.^19^ In a meta-analysis by Busquets and Hwang,^20^ the mean recurrence rate for the endoscopic surgery, taking all stages together, was 15% vs. 20% for external approaches (*p* = 0.001).

However, it should be borne in mind that cases with more advanced disease – selected Krouse T3 and especially Krouse T4 IP – are those most often indicated for an open or combined approach. The analysis of the subset of Krouse T3 and T4 IP showed that the recurrence rates among the surgical groups continued not to be statistically relevant (*p* = 0.580) (Table 4). Nevertheless, a critical interpretation should be employed in the analysis of these results given the smaller sample of T3 and T4 Krouse IP.

Contradictory results have been reported regarding the ability of the Krouse classification system to estimate recurrence. Some studies highlighted an association between recurrence and the Krouse classification,^21^ while others did not.^4^, ^22^, ^23^ In our perspective, Krouse classification may include some limitations in predicting the IP recurrence.

In particular, T3 Stage gathers IP with notable location heterogeneity like the anterior maxillary sinus wall, sphenoid sinus or frontal sinus. In fact, the site of origin in the frontal sinus has been consistently pointed as a risk factor for recurrence.^14^, ^19^ Krouse T3 Staged IP were preferably treated by endoscopic techniques and presented an overall recurrence rate of 57.1% which corresponded to 80% of all tumor relapses. This finding is in accordance with a series of more than 500 cases reported by Kim et al.^14^ in which the endoscopically managed T3 Stage IP revealed higher rates of recurrence. In the current review, the greater recurrence rate observed in T3 tumors could be related to the endoscopic approach of some cases that might well have a better indication for combined surgery.

The critical steps for the IP resection consist in a clearly identification of the implantation site, careful removal of the mucosal rim and complementary drilling of the underlying bone. Despite the impressive evolution of the endoscopic techniques and indications, we believe that there is still a role for combined and open approaches in selected cases of IP. Whenever the tumor attachment is far lateral or anterior in the maxillary sinus or too lateral in the frontal sinus, endoscopic surgery may be complemented with an external surgery such as a Caldwell-Luc approach or a lateral rhinotomy, respectively. Regarding the specifity of the frontal sinus origin, Walgama and coworkers^24^ referred lower rates of recurrence in those patients treated with a more aggressive surgical technique such as the osteoplastic approach or the endoscopic modified Lothrop procedure. Similarly, a combined approach may be favored whenever the orbital or intracranial compartments are involved.

Some reports^4^, ^5^, ^25^ have found that IP tend to relapse more often during the first two years after surgery, as happened in our review (Fig. 1). Sham et al.^4^ found that 67%–72% of the first recurrences were discovered within the first year of follow-up and 83%–89% within the first two years of follow-up. In the largest published series to date, Kim and coworkers^14^ reported a mean recurrence time of 32.6 months. These results suggest that these early recurrences of IP might be better related to a persistence of the disease, probably explained by the presence of tumor remnants in the improperly drilled bone or an inadequate identification of the implantation site. Although most early recurrences are likely to be attributable to residual diseases, a few late recurrences, particularly those occurring at separate new foci, may represent true recurrences or multifocal tumors.^4^

Mortality in IP with associated carcinoma, whether synchronous or metachronous, is not negligible. A meta-analysis found a 126 months median survival; and a 3 year survival rate of 63%.^26^ The rate of malignancy in our series of IP was 2.7% corresponding to a synchronous SCC which is a lower incidence than previous reports with rates between 3.8% and 6.6%.^14^, ^20^ Although there are no validated protocols yet, it has been our option to treat cases of concurrent SCC with surgery while adjuvant RT should be considered. A retrospective study of 32 cases of IP with malignant transformation concluded that surgery followed by RT was more effective than exclusive surgery or RT in terms of 5 year survival, which was 84% for associated versus 41% for exclusive treatment (*p* = 0.006).^27^

The current series, though numerically limited, establishes the role of the endoscopic surgery as an oncological safe technique, whereas the classic open or combined approaches may be best suited for some specific T3 and T4 Krouse IP. Moreover, it emphasises that IP persistence may be more relevant than IP recurrence. It is important to note that this study focused on the Krouse classification system and on the different surgical approaches as potential risk factors for recurrence. However, other factors such as drilling the underlying bone or the HPV status are still debated as potential risk factors for recurrence.^28^, ^29^ Future research with prospective studies, systematic reviews and meta-analyses may be helpful to clarify the potential role of these factors on recurrence rate.

## Conclusion

Endoscopic surgery did not increase the recurrence rates and can be a safe and efficient alternative to open or combined techniques for IP treatment. The recurrence of inverted papilloma seem to be related to the persistence of the disease and tend to occur early after primary surgery. Krouse T3 Stages may be associated with a higher recurrence of IP. The advent of the endoscopic surgery, with new techniques and more angulated instruments, could potentially increase the access to the more difficult areas such as the frontal sinus and the anterior and lateral maxillary sinus walls, further improving surgical outcomes.

## Conflicts of interest

The authors declare no conflicts of interest.
